# Epigallocatechin-3-*O*-gallate up-regulates microRNA-let-7b expression by activating 67-kDa laminin receptor signaling in melanoma cells

**DOI:** 10.1038/srep19225

**Published:** 2016-01-12

**Authors:** Shuhei Yamada, Shuntaro Tsukamoto, Yuhui Huang, Akiko Makio, Motofumi Kumazoe, Shuya Yamashita, Hirofumi Tachibana

**Affiliations:** 1 Division of Applied Biological Chemistry, Department of Bioscience and Biotechnology, Faculty of Agriculture, Kyushu University, Fukuoka 812-8581, Japan

## Abstract

MicroRNAs (miRNAs) are non-coding RNAs involved in various biological processes by regulating their target genes. Green tea polyphenol (−)-epigallocatechin-3-*O*-gallate (EGCG) inhibits melanoma tumor growth by activating 67-kDa laminin receptor (67LR) signaling. To examine the effect of EGCG on miRNA expression in melanoma cells, we performed miRNA microarray analysis. We showed that EGCG up-regulated miRNA-let-7b expression through 67LR in melanoma cells. The EGCG-induced up-regulation of let-7b led to down-regulation of high mobility group A2 (HMGA2), a target gene related to tumor progression. 67LR-dependent cAMP/protein kinase A (PKA)/protein phosphatase 2A (PP2A) signaling pathway activation was involved in the up-regulation of let-7b expression induced by EGCG. These findings provide a basis for understanding the mechanism of miRNA regulation by EGCG.

MicroRNAs (miRNAs) are functional, small, non-coding RNAs that regulate target gene expression by inducing mRNA degradation or translational inhibition^1^. Numerous studies have shown that miRNAs play a critical role in many biological processes such as cell proliferation^2^, apoptosis^3^, differentiation^4^, inflammation^5^ and metabolism^6^. Thus, miRNAs have been proposed to be related to various diseases^7^,^8^. It has been reported that some miRNAs are abnormally expressed in cancer cells and their dysregulation leads to cancer progression^9^,^10^,^11^. For example, miR-21 is overexpressed in many types of cancer and its down-regulation inhibits non-small cell lung cancer cell proliferation and migration^12^. Therefore, modulation of miRNA activity could be an effective strategy for cancer therapy.

(−)-Epigallocatechin-3-*O*-gallate (EGCG) is a major polyphenolic compound in green tea extracts. Numerous studies have shown that EGCG has anti-cancer activities^13^,^14^ and its potential value as an anti-cancer agent has been demonstrated in several human investigations^15^,^16^,^17^. The 67-kDa laminin receptor (67LR) has been identified as a cell surface receptor of EGCG^18^ and plays a key role in the cancer preventive effects of EGCG^19^,^20^,^21^,^22^. In melanoma, the most aggressive form of skin cancer, 67LR is expressed at a higher level than normal skin cells^23^. We have previously shown that EGCG suppresses melanoma tumor growth by activating the intercellular signaling pathway, cAMP/protein kinase A (PKA)/protein phosphatase 2A (PP2A), as an agonist of 67LR^23^.

Several studies have revealed that dietary polyphenols have the potential to modulate miRNA expression^24^,^25^,^26^,^27^. The modulation of miRNAs by polyphenols may be partially relevant to their physiological effects. However, the effects of EGCG on miRNA expression in melanoma cells are unknown.

In this study, we aimed to elucidate the effect of EGCG on miRNA expression in melanoma cells and the mechanism of miRNA regulation induced by EGCG. We performed comprehensive analysis of miRNA expression profiles when melanoma cells were treated with EGCG. Moreover, we assessed the involvement of 67LR signaling molecules in the miRNA regulation mechanism of EGCG.

## Results

### MiRNA expression profiles induced by EGCG through 67LR in melanoma cells

**Figure 1:**
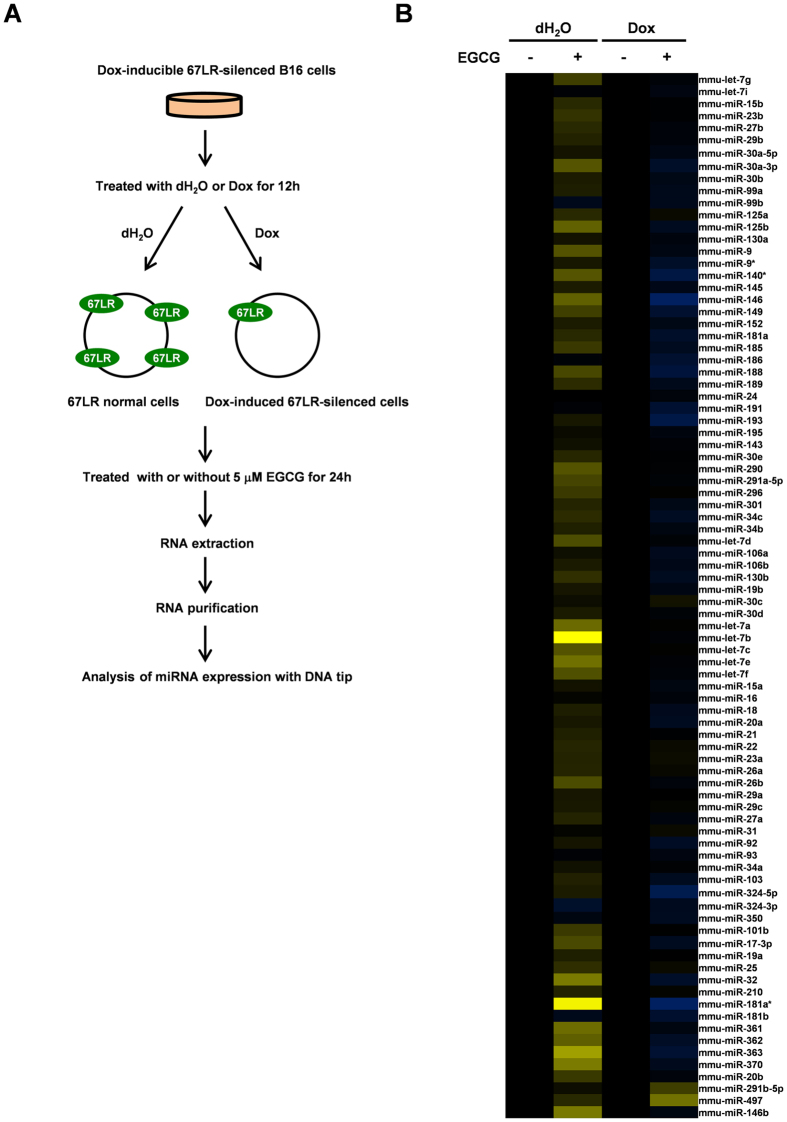
Comprehensive analysis of miRNA expression affected by EGCG through 67LR. (**A**) B16 cells were treated with dH_2_O or doxycycline (Dox) for 12 h to reduce 67LR expression and then cells were treated with or without EGCG for 24 h. After purification of small RNAs, miRNA expression was measured by DNA tip. (**B**) Results were shown as relative to each EGCG-untreated cell. Each control was shown as black bar, high; yellow, low; blue.

EGCG exerts anti-melanoma activity in a 67LR-dependent manner^20^,^23^. To examine the effect of EGCG on miRNA expression in mouse melanoma B16 cells and the involvement of 67LR, we performed miRNA microarray analysis. Expression of some miRNAs was up-regulated by EGCG, and doxycycline-induced 67LR silencing attenuated the effect of EGCG (Fig. 1A,B and Supplementary Table). By using quantitative real-time PCR analysis, we examined the effect of EGCG on let-7a, let-7b, let-7e, miR-146b and miR-26a expression. EGCG up-regulated let-7a, let-7b, let-7e and miR-146b expression, whose expressions were up-regulated in miRNA microarray analysis (Supplementary Fig. 1). On the other hand, miR-26a expression was not affected by EGCG (Supplementary Fig. 2). Among these miRNAs, we focused on miRNA-let-7b whose expression was significantly up-regulated by EGCG.

### EGCG increases let-7b expression through 67LR in melanoma cells

**Figure 2:**
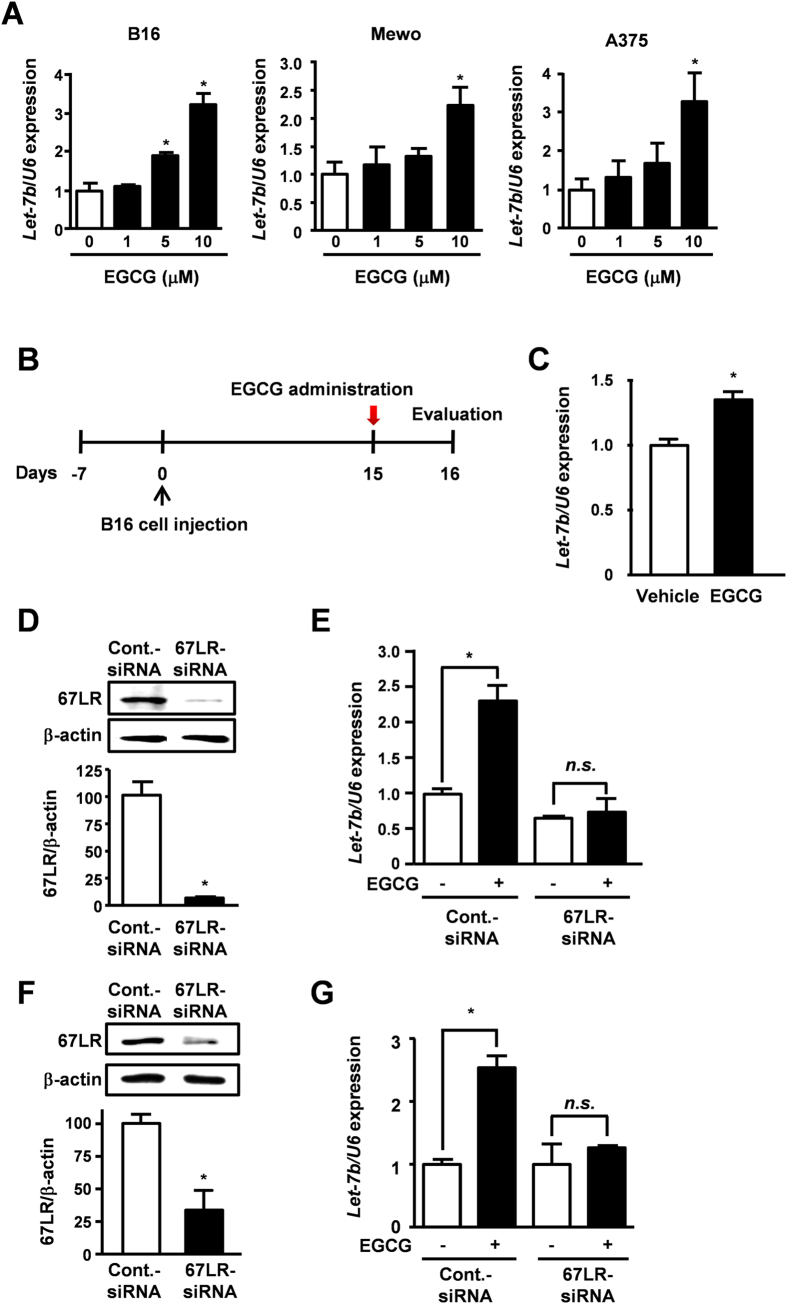
EGCG increases let-7b expression through 67LR in melanoma cells. (**A**) B16, Mewo and A375 cells were treated with indicated concentrations of EGCG for 24 h, and then let-7b expression was measured by q-RT-PCR. (**B**,**C**) 15 days after tail vein injection of B16 cells, mice were administered with EGCG intraperitoneally (20 mg/kg b.w.) or by vehicle. 24 h after administration, let-7b expression of lung metastatic tumor was measured. Mean ± S.E. *N* = 4. (**D**,**F**) 67LR protein expression in B16 or Mewo cells transfected with 10 nM 67LR-siRNA for 72 h. (**E**,**G**) Cells transfected with 10 nM 67LR siRNA for 48 h were treated with 10 μM EGCG for 24 h. Let-7b expression was measured. Mean ± S.E.

By using quantitative real-time PCR analysis, we confirmed that EGCG dose-dependently increased let-7b expression in three melanoma cell lines, B16, Mewo and A375 (Fig. 2A). We also examined the effect of EGCG on let-7b expression in metastatic melanoma tumors (Fig. 2B). Single administration of EGCG increased let-7b expression in lung metastatic melanoma tumor cells (Fig. 2C). To assess the involvement of 67LR in this effect, 67LR expression in B16 and Mewo cells was silenced by introducing siRNA specifically targeting 67LR (Fig. 2D,F). The expression of let-7b was not significantly affected by EGCG in 67LR-silenced cells (Fig. 2E,G). These results showed that EGCG up-regulated let-7b expression through 67LR in melanoma cells.

### EGCG decreases HMGA2 expression by modulating let-7b activities

**Figure 3:**
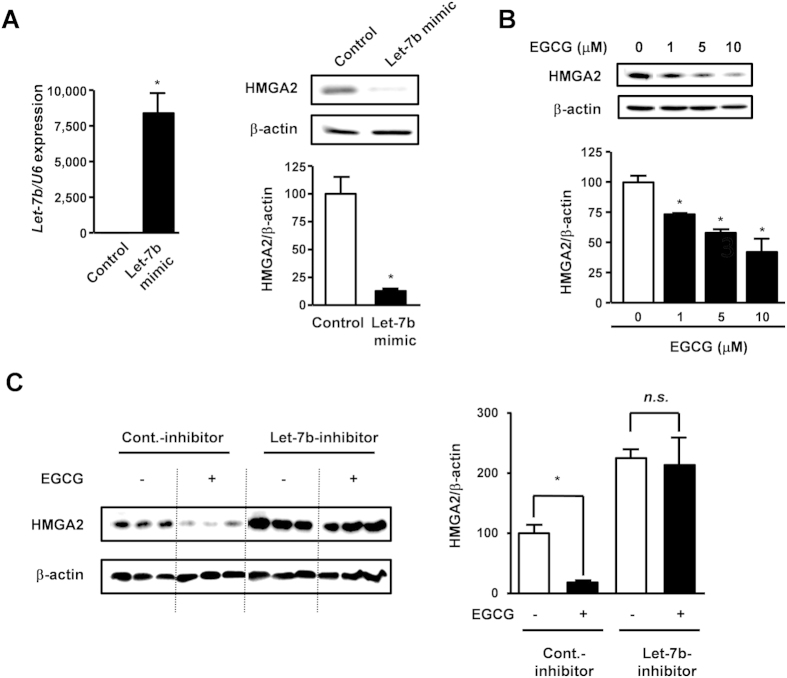
EGCG suppresses HMGA2 expression by modulating let-7b activities. (**A**) Let-7b expression or HMGA2 expression in B16 cells transfected with 10 nM let-7b mimic. (**B**) HMGA2 expression in B16 cells treated with EGCG for 72 h. (**C**) HMGA2 expression was evaluated when B16 cells were transfected with 50 nM let-7b inhibitor for 48 h and then treated with 10 μM EGCG for 72 h. Mean ± S.E.

Let-7b has several target genes related to tumor progression including HMGA2 (high mobility group A2). HMGA2 has been reported to be highly expressed in tumors^28^ and promotes tumor progression in several types of tumor, for example oesophageal squamous cell carcinomas^29^. We examined whether let-7b down-regulates HMGA2 expression in melanoma cells. The transfection of let-7b mimic decreased HMGA2 protein expression in melanoma cells (Fig. 3A). EGCG dose-dependently decreased HMGA2 expression (Fig. 3B). To assess whether the up-regulation of let-7b elicited by EGCG was involved in this effect, we used anti-let-7b nucleotide, which inhibits endogenous let-7b activities. EGCG decreased HMGA2 expression in control cells, whereas let-7b inhibition attenuated the effect of EGCG (Fig. 3C). These results demonstrated that EGCG decreased HMGA2 expression by modulating let-7b expression.

### Activation of the cAMP/PKA/PP2A signaling pathway elicits up-regulation of let-7b expression

**Figure 4:**
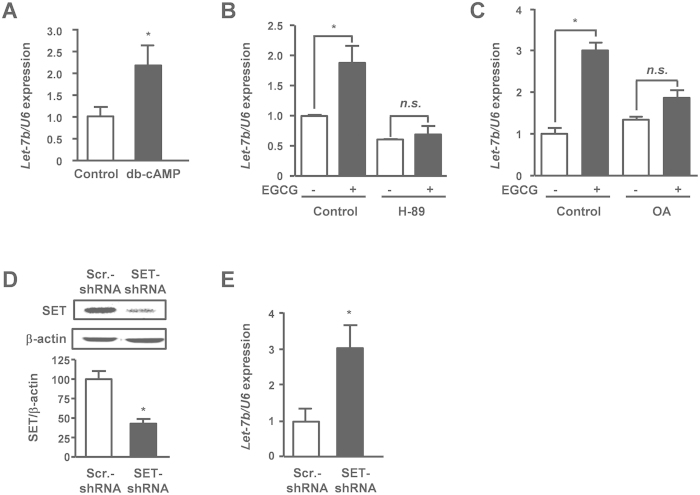
Activation of cAMP/PKA/PP2A signaling pathway induces the up-regulation of let-7b expression. (**A**) Let-7b expression in B16 cells treated with 2 mM dibutylyl-cAMP (db-cAMP). (**B**) Let-7b expression in B16 cells treated with 10 μM EGCG, and 10 μM H-89 separately or in combination for 24 h. (**C**) Let-7b expression in B16 cells treated with 10 μM EGCG, and 5 nM okadaic acid (OA) separately or in combination for 24 h. (**D**,**E**) Let-7b expression in SET-silenced B16 cells was measured by q-RT-PCR. Mean ± S.E.

In our previous study, we demonstrated that EGCG suppresses melanoma cell proliferation through 67LR following activation of the cAMP/PKA/PP2A signaling pathway^23^. To elucidate the mechanism of let-7b up-regulation induced by EGCG via 67LR, we examined the involvement of these 67LR signaling molecules. Dibutyryl-cAMP (db-cAMP), an analogue of cAMP, increased let-7b expression (Fig. 4A). To examine the involvement of PKA and PP2A, melanoma cells were co-treated with EGCG and each specific inhibitor. H-89 (PKA inhibitor) and OA (PP2A inhibitor) attenuated the effect of EGCG on let-7b expression (Fig. 4B,C). SET (Suver3-9, enhancer of zeste, trithorax), which is overexpressed in melanoma cells, inhibits the EGCG-induced PP2A activation^23^. To examine the effect of PP2A activation on let-7b expression, we constructed SET-silenced cells (Fig. 4D). The expression of let-7b in SET-silenced cells was significantly higher than that in control cells (Fig. 4E). These results indicated that activation of the cAMP/PKA/PP2A signaling pathway was involved in the let-7b up-regulation induced by EGCG in melanoma cells.

## Discussion

The objective of this study was to elucidate the effect of EGCG on miRNA expression in melanoma cells and the mechanism of miRNA regulation induced by EGCG. EGCG increased let-7b expression through 67LR. We found that EGCG up-regulated let-7b expression not only in melanoma cell lines but also in metastatic melanoma tumors *in vivo*. Moreover, we demonstrated that 67LR signaling pathway activation was involved in the up-regulation of let-7b elicited by EGCG.

Excluding the miRNAs which were not detected, we analyzed expression of 86 miRNAs in our microarray analysis. Interestingly, the effect of EGCG on miRNA expression in 67LR-silenced melanoma cells was exclusively different from that in 67LR normal melanoma cells. These results suggest that 67LR plays a key role in EGCG-induced miRNA regulation in melanoma cells. Zhou *et al.* reported that administration of EGCG up-regulates miR-210 expression in the NNK-induced A/J mouse lung tumor^27^. However, miR-210 expression was not affected by EGCG in our melanoma-based microarray analysis. Further studies on other types of cancer or *in vivo* experiments are needed to elucidate the effect of EGCG on miRNA expression in cancer.

EGCG decreased HMGA2 expression by modulating let-7b activities. In addition to HMGA2, let-7b has multiple target genes related to tumor progression such as Ras^30^,^31^. Therefore, the EGCG-induced up-regulation of let-7b may influence its target genes and may repress melanoma tumor progression pathways.

Our previous study found that EGCG activates the 67LR signaling pathway in melanoma cells as follows^23^. EGCG induces intracellular cAMP production and then activates PKA through 67LR. The EGCG-induced PP2A activation is mediated by the cAMP/PKA pathway. These 67LR signaling pathway has a key role for the anti-melanoma effect of EGCG.

We also demonstrated that PP2A activation induced the up-regulation of let-7b expression. PP2A is a major serine-threonine phosphatase that suppresses tumor progression by regulating multiple intercellular signaling driven by protein kinases^32^. It has been reported that PP2A is frequently inactivated in cancer cells and loss of functions of PP2A leads to tumor progression^33^. Furthermore, let-7b is abnormally down-regulated in many types of cancer including melanoma. Our results suggest that dysregulation of let-7b expression is attributed to PP2A inactivation in melanoma. Since regulation mechanisms for expression of individual miRNA molecules are largely unknown, the mechanism of let-7b regulation demonstrated in this study contributes to understanding of other miRNA regulation mechanisms.

In most cases, miRNAs are transcribed from their genes as primary miRNA (pri-miRNA). Drosha, a class 2 RNA III enzyme, processes pri-miRNA to a precursor miRNA (pre-miRNA) in the nucleus. In the cytoplasm, mature miRNAs are synthesized from pre-miRNA by Dicer^34^. It is still unclear whether EGCG-induced PP2A activation promotes let-7b transcription or positively regulates let-7b processing. Further studies are needed to elucidate the mechanism between PP2A activation and let-7b up-regulation.

Based on the 67LR signaling pathway, our study revealed a novel mechanism of let-7b regulation in melanoma cells. These data help us to further understand a relationship between EGCG and functional miRNAs.

## Methods

### Cell lines, reagents and antibodies

Mouse melanoma B16, human melanoma Mewo and A375 cells were purchased from the American Type Culture Collection (ATCC) and were maintained in Dulbecco’s modified Eagle’s medium (DMEM, Gibco, CA, USA) containing 5% (for B16 cells) or 10% (for other cells) fetal bovine serum (FBS, Gibco). All cells were in a state of logarithmic growth at 37 °C in a humidified chamber with 5% CO_2_. To examine the effects of EGCG, cells were harvested from culture plates and treated with EGCG at the indicated concentrations for the indicated time in DMEM supplemented with 1% FBS including 200 units/mL catalase (Sigma) and 5 units/mL superoxide dismutase (Sigma).

EGCG, catalase, H-89, okadaic acid, dibutyryl-cAMP and the anti-β-actin antibody were purchased from Sigma-Aldrich. Anti-HMGA2 antibody was obtained from Cell Signaling Technology (Beverly MA). Anti-67LR serum was obtained from a rabbit, which had been immunized with synthesized peptides corresponding to residues 161-170 of human 67LR.

Mmu-67LR-siRNA (#4390771), hsa-67LR-siRNA (#4392420) and negative control-siRNA (#4390843) were purchased from Ambion (Austin, TX, USA). Mission microRNA mimic of hsa-let-7b-5p (HMI0007), Mission synthetic microRNA inhibitor of hsa-let-7b-5p (HSTUD0007) and negative control nucleotides (HMC0003 and #199004-00) were purchased from Sigma Aldrich.

### Construction of doxycycline-inducible 67LR-silenced cells

The procedure for construction of doxycycline-induced 67LR-silenced cells was described previously^23^.

### Microarray analysis

B16 cells were treated with 5 μM EGCG for 24 h and total RNAs were extracted with TRizol reagent (Invitrogen, Carlsbad, CA, USA) according to the manufacturer’s instructions. Then, RNAs were purified using RNeasy MinElute Kit (Qiagen) according to the manufacturer’s instructions. Expression levels of miRNA were measured by DNA tip genopal MICM (Mitsubishi Reyon, Tokyo, Japan).

### Measurement of let-7b expression in lung metastatic melanoma tumor

5-week-old female C57BL/6J mice were purchased from Kyudo Company (Saga, Japan). Mice were injected into the tail vein with 5 × 10^5^ B16 cells in 100 μL PBS. 15 days after injection, vehicle alone or EGCG (20 mg/kg body weight) were administered intraperitoneally. 24 h after administration, RNAs of lung metastatic colonies were extracted. This experiment was carried out according to the guidelines for animal experiments at the Faculty of Agriculture and Graduate Course, Kyushu University, and Law (No. 105) and Notification (No. 6) of the Japanese government. All animal experiments were approved by the Animal Care and Use Committee of Kyushu University, Fukuoka, Japan (approval number A26-090-7).

### Real-time quantitative RT-PCR (q-RT-PCR)

Total RNAs were extracted with TRizol reagent (Invitrogen). q-RT-PCR was performed using the miRCURY LNA^TM^ Universal RT microRNA PCR system (Exiqon) according to the manufacturer’s instructions. Briefly, total RNA was reverse transcribed to cDNA using Universal cDNA synthesis kit II (Exiqon). q-RT-PCR was performed using the ExiLENT SYBR Green master mix (Exiqon) and CFX96 Touch^TM^ Real Time PCR Detection System (Bio-Rad). LNA^TM^ PCR primer mix; hsa-let-7b (#205727) was purchased from Exiqon. In the experiments presented here, miRNA expression was normalized to U6 small nuclear RNA (Exiqon, #203907).

### RNA transfection

RNA reagents were introduced into cells with Lipofectamine^TM^ RNAiMAX (Ambion, Austin, TX, USA) according to the manufacturer’s instructions. Briefly, RNA reagents, RNAiMAX and medium were mixed gently with pipetting. After leaving for 10 min at room temperature, the complexes were added to the cells and cultured for the indicated time.

### Western blotting

Cells were lysed in lysis buffer containing 50 mM Tris-HCl (pH 7.5), 150 mM NaCl, 1% Triton X-100, 1 mM ethylenediamine tetra-acetic acid (EDTA), 50 mM sodium fluoride (NaF), 30 mM sodium pyrophosphate (Na_4_P_2_O_7_), 1 mM phenylmethanesulfonyl fluoride (PMSF), and 2 mg/mL aprotinin. Approximately 50 μg of protein was suspended in Laemmli sample buffer (0.1 M Tris-HCl buffer, pH 6.8, 1% SDS, 0.05% mercaptoethanol, 10% glycerol and 0.001% bromophenol blue), boiled and electrophoresed on 8% SDS-polyacrylamide gels. Gels were then electroblotted onto Trans-Blot nitrocellulose membranes (Bio-Rad). Incubation with the indicated antibodies was performed in Tween 20/PBS (TPBS) containing 1% bovine serum albumin (BSA). Blots were washed with TPBS and incubated with anti-rabbit or anti-mouse horseradish peroxidase (HRP) conjugates. After washing, specific proteins were detected using an enhanced chemiluminescence system according to the manufucturer’s instructions (Amersham Biosciences).

### SET silencing by shRNA

Lentiviral vectors expressing non-targeting scramble shRNA (Scr-shRNA) and shRNA targeting SET were purchased from Sigma-Aldrich. Lentivirus production, transduction and selection were performed according to the manufacturer’s instructions.

### Statistical analysis

Data were analyzed with GraphPad Prism (version 4) using Student’s *t*-test when comparing two conditions, Dunnett’s test when comparing with control or Tukey’s test for multiple comparisons. Values of *P* < 0.05 represented with an asterisk were considered significant.

## Additional Information

**How to cite this article**: Yamada, S. *et al.* Epigallocatechin-3-*O*-gallate up-regulates microRNA-let-7b expression by activating 67-kDa laminin receptor signaling in melanoma cells. *Sci. Rep.* **6**, 19225; doi: 10.1038/srep19225 (2016).

## Supplementary Material

Supplementary Figure

Supplementary Table
