# Supplementary material for: Epigallocatechin-3-O-gallate up-regulates microRNA-let-7b expression by activating 67-kDa laminin receptor signaling in melanoma cells
Source: Sci Rep. 2016 Jan 12;6:19225. doi: 10.1038/srep19225 (PMC4709792; doi:10.1038/srep19225)
Supplement: Supplementary Figure [file srep19225-s1.pdf]

Epigallocatechin-3-*O*-gallate up-regulates microRNA-let-7b expression by activating 67-kDa laminin receptor signaling in melanoma cells

Shuhei Yamada, Shuntaro Tsukamoto, Yuhui Huang, Akiko Makio, Motofumi Kumazoe, Shuya Yamashita & Hirofumi Tachibana

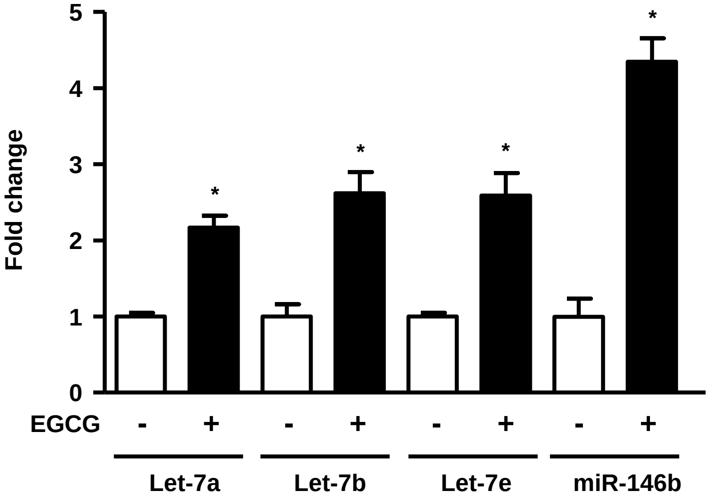

Supplementary Figure 1: EGCG up-regulates several miRNAs expression.
B16 cells were treated with 10 μM EGCG for 24 h, and then miRNAs expression was measured by q-RT-PCR.

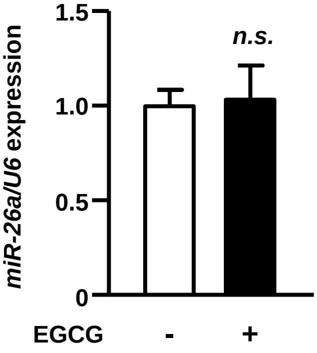

Supplementary Figure 2: miR-26a expression was not affected by EGCG.
B16 cells were treated with 10 μM EGCG for 24 h, and then miR-26a expression was measured by q-RT-PCR.
